# *B3GNT2* , *GPR35* , *PSMG1* Gene Polymorphisms Are Related With Susceptibility and Severity of Ankylosing Spondylitis in Chinese Han Population

**DOI:** 10.1002/mgg3.70125

**Published:** 2025-07-30

**Authors:** Zijian Lian, Bin Zhao, Wei Luo, Jun Liu, Jing Wang, Wei Chai, Yan Wang, Songqing Ye, Xinlong Ma

**Affiliations:** ^1^ Department of Orthopaedics Tianjin Hospital Tianjin University Tianjin China; ^2^ Department of Orthopaedics Chinese People's Liberation Army General Hospital Beijing China

**Keywords:** AS, *B3GNT2*, Chinese Han population, *GPR35*, polymorphism, *PSMG1*, SNP

## Abstract

**Background:**

Latest research on ankylosing spondylitis (AS) indicates a link between the *B3GNT2*, *PSMG1* genes and susceptibility to AS among western populations. However, the association of these three genes with AS in eastern populations remains insufficiently explored. It is necessary to replicate these studies in other populations. Consequently, we chose tagSNPs in these three genes in the Chinese Han population to be sequenced.

**Purpose:**

We tried to find the SNP loci that are associated in both eastern and western populations through repeated experiments. Furthermore, our research extended to examining the link between these genes and the severity of AS. This study aimed to evaluate the association between the tagSNPs of B3GNT2 (rs10865331, rs6545925, rs467250), the rs4676410 SNP on *GPR35*, and the rs4816648 SNP of *PSMG1* with AS susceptibility and disease activity in a Chinese Han population.

**Method:**

We collected blood samples from 497 patients with AS and 498 control subjects and sequenced 5 tagSNPs in *B3GNT2*, 1 tagSNP in *GPR35*, and 6 tagSNPs in *PSMG1*.

**Result:**

Within the five selected tagSNPs of *B3GNT2*, the rs10865331, rs6545925, and rs4672501 tagSNPs are associated with susceptibility to AS. Additionally, the rs4672501 SNP is not only associated with susceptibility to AS, but also with the severity of AS. For the first time, we find that the rs4676410 SNP on the *GPR35* gene is associated with susceptibility to AS, but not associated with the severity of AS in the Chinese Han population. We find for the first time that the rs4816648 SNP of the *PSMG1* gene is associated with both susceptibility and severity of ankylosing spondylitis.

**Conclusion:**

*B3GNT2 and PSMG1* genes are related to both susceptibility and severity of AS. The *GPR35* gene is related to susceptibility to AS in the Chinese Han population, which corroborates the findings of research conducted in western populations.

## Graphical abstract

*B3GNT2*, *GPR35*, and *PSMG1*genes are related to ankylosing spondylitis in the Chinese Han population.

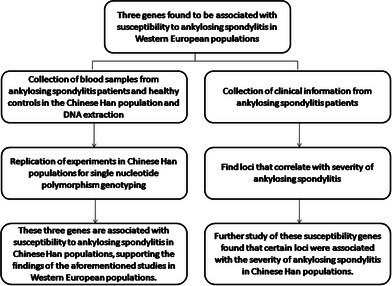

## 1 Introduction

AS is a persistent inflammatory condition that initially presents as sacroiliac arthritis. This condition ultimately results in the merging of the spine and joints, along with various other disorders, profoundly impacting the patient's quality of life (Ashrafi et al. 2020). This ailment has a strong link to the HLA‐B27 gene, yet only 1%–5% of those testing positive for HLA‐B27 exhibit AS. This condition is marked by the swelling of the spine and sacroiliac joints, leading to discomfort, rigidity, and ultimately, the development of new bones and gradual joint ankylosis. Increasing evidence suggests the involvement of additional genes (Brown et al. 2000). Genetic factors primarily dictate the severity of AS. While the majority of genetic research has concentrated on disease susceptibility, the severity of AS has been the subject of limited studies (Hamersma et al. 2001).

The genes studied in the present report include *B3GNT2*, *GPR35*, and *PSMG1*. *B3GNT2*, known as beta‐1,3‐N‐acetylglucosaminyltransferase 2, had the ability to both start and extend poly‐N‐acetyllactosamine chains, confirming its similarity to the poly‐N‐acetyllactosamine synthase enzyme (Zhou et al. 1999). *GPR35* (G protein‐coupled receptor 35) manifested in mucosal tissues, dendritic cells, macrophages, and granulocytes, triggering chemotactic reactions in a human monocyte cell line, and was communicated via a new chemokine receptor (Maravillas‐Montero et al. 2015). *PSMG1* (proteasome assembly chaperone 1) was found by Hirano et al. (2005), and the authors determined the mechanism by which the 20S proteasomes are correctly assembled.

In the study of AS susceptibility among western populations, the rs10865331 SNP in *B3GNT2* emerged as more significant than all other SNPs. Conversely, the SNP rs2242944 close to *PSMG1* shows a significant correlation with AS (Australo‐Anglo‐American Spondyloarthritis Consortium (TASC) et al. 2010). A link was established between the rs4676410 SNP located on *GPR35* and the onset of IBD (Kirkik et al. 2025). A variety of genetic sites linked to IBD are also connected to different autoimmune disorders, notably AS and psoriasis (Jostins et al. 2012). We divided AS patients into severe AS and normal AS groups according to the severity of the patients. We further investigated the relationship between the selected tagSNPs and the severity of AS. In order to study the severity of the disease, we divided the case group into a severe group and a normal group and verified that there was a difference between these two groups using clinical scoring methods such as BASFI (Bath AS function index), BASDAI (Bath AS disease activity index) and the mSASSS (The modified Stokes AS Spine Score). The research we conducted can analyze the parallels and variances among these three genes between the western and eastern populations. The discovery of new susceptibility loci and the derivation of alternative loci in relation to the severity of AS will provide sufficient evidence for the study of the pathogenesis of AS.

## 2 Methods

### 2.1 Study Population

The research involved enlisting 497 patients with AS and 498 unrelated healthy individuals, aligned by age and gender. Recruitment for this research spanned from January 1, 2016, to January 1, 2023. Every patient and control had Han Chinese ancestry. Every AS patient was tested positive for HLA‐B27. All AS patients consistently received non‐steroidal anti‐inflammatory medications, while no alternative treatments were employed by these individuals. Patients with AS comprised 439 men (88.3%) and 58 women (11.7%), averaging 31.21 years in age (spanning 16 to 60 years) (Table 1). Comprising the control group were 431 males (86.5%) and 67 females (13.5%), averaging 30.6 years in age (spanning from 16 to 60 years). No significant differences were noted in gender (*p* = 0.396) or age (*p* = 0.327) between the AS group and the control group. The typical length of time since being diagnosed with AS was 10.6 years (8 to 18 years). Skilled rheumatologists confirmed the diagnosis of AS. All diagnoses were made according to the modified New York criteria (van der Linden et al. 1984). Subjects with conditions such as inflammatory bowel disease, psoriasis, rheumatoid arthritis, or other autoimmune disorders were excluded from both the AS and control groups.

**TABLE 1:** Demographic data of AS patients and controls.

		Cases (497)	Controls (498)	*p*
Sex	Male	439 (88.330%)	431 (86.546%)	0.396
	Female	58 (11.670%)	67 (13.454%)	
Age		31.171 ± 8.470	30.653 ± 8.218	0.327
Duration of diagnosis		10.612 ± 2.978	N/A	
BASFI		4.296 ± 1.749	N/A	
BASDAI		4.195 ± 1.188	N/A	
mSASSS		16.394 ± 16.405	N/A	

*Note:* There is no significant difference in age and sex‐distribution between AS patients and controls. Numerical values presented as mean ± standard deviation.

Abbreviations: BASDAI, bath ankylosing spondylitis disease activity index; BASFI, bath ankylosing spondylitis function index; mSASSS, modified Stokes ankylosing spondylitis Spine Score.

### 2.2 Obtaining Clinical Data and Classification by Severity

The questionnaires BASFI and BASDAI were distributed to patients. The primary technique for assessing AS functional status and disease activity involves the use of these indices (Calin et al. 1994; Garrett et al. 1994). The mSASSS, a well‐established scoring technique, is used to assess persistent spinal changes (Baraliakos et al. 2009; Sieper et al. 2009; Creemers et al. 2005). Each participant underwent standard anteroposterior and lateral X‐rays of their cervical and lumbar spine, and the mSASSS score for each was computed from this lateral viewpoint. The mSASSS scores were individually allocated by three authors, with the mean score being utilized. Agreement is lacking on the categorization of AS severity (Amor et al. 1994). This research characterizes severe AS as a disease type in patients needing surgical intervention within a decade post‐diagnosis. Surgical signs encompass difficulties in standing, forward gaze, and viscera compression caused by kyphosis, resulting in pain (Kiaer and Gehrchen 2010). Patients with normal AS exhibit inflammation in the sacroiliac joint, but their spinal and other joints are mostly unaffected, requiring only medical treatment and avoiding surgery within a decade of being diagnosed. According to the given definition, 164 patients with AS were classified as severe subtypes, while 333 patients with AS were considered normal subtypes. Table 2 displays a comparative analysis of clinical characteristics between severe AS and normal AS.

**TABLE 2:** Clinical features comparing severe AS and normal AS.

		Severe AS (164)	Normal AS (333)	*p*
Sex	Male	140 (85.366%)	299 (89.790%)	0.149
	Female	24 (14.634%)	34 (10.210%)	
Age		31.732 ± 8.955	30.895 ± 8.220	0.315
Duration of diagnosis		10.671 ± 4.505	10.583 ± 1.811	0.810
BASFI		6.029 ± 2.046	3.442 ± 0.542	< 0.001
BASDAI		5.488 ± 1.088	3.559 ± 0.544	< 0.001
mSASSS		37.073 ± 13.210	6.210 ± 1.066	< 0.001

*Note:* There is no difference between severe AS patients and normal patients in sex distribution, age and duration of diagnosis; however, the BASFI, BASDAI and mSASSS are higher in severe AS patients.

### 2.3 SNPs Selection

The SNPs investigated in this study include 5 tagSNPs in *B3GNT2*, 1 tagSNP in *GPR35*, and 6 tagSNPs in *PSMG1* that were sequenced. *B3GNT2* and *GPR35* localize to chromosome 2. *PSMG1* localizes to chromosome 21. The selected SNPs served as a multi‐marker tagging algorithm with criteria of r^2^ more than 0.8 and for all SNPs with minor allele frequency more than 5% from the Han Chinese in Beijing (CHB) population in the HapMap database. Haploview 4.2 software (Broad Institute, Cambridge, Massachusetts, USA) was used to select the tagSNPs. Figure S1 illustrates the placements of each chosen tagSNP on the genes. The SNP rs4672482 is in exon 2 of *B3GNT2*. The SNP rs4676410 is in exon 6 of *GPR35*. The SNP rs2242944 is near the promoter of *PSMG1*. Other SNPs are all in the introns of their respective genes.

### 2.4 DNA Extraction and Genotyping Analysis

Using the AxyPrep Blood Genomic DNA Miniprep kit (Axygen Biosciences, Union City CA), DNA was extracted from 2 mL samples of whole blood. The MassARRAY system (Sequenom, San Diego, CA, USA) was used to identify SNPs. The study employed matrix‐assisted laser desorption/ionization time‐of‐flight mass spectrometry (MALDI‐TOF‐MS). The majority of SNPs underwent successful genotyping. The identification of genotypes exceeded 98% in each of the case and control groups.

### 2.5 Statistical Analysis

We estimated the sample size using the following formula: *n* = [2 × *p* × (1 − *p*) × (*Z*_*α*_ + *Z*_*β*_)^2^]/(*p*1 − *p*2)^2^. “*n*” was sample size in each group. Power was 0.8, *α* = 0.05, *Z*_*α*_ + *Z*_*β*_ = 1.96 + 1.28, *p*1 denoted the rate of one of the genotypes in the case group, *p*2 denoted the rate of this genotype in the control group, and *p* denoted the mean of the rates in the above two. Each of the 12 tagSNPs underwent testing to determine the Hardy–Weinberg equilibrium. To assess the differences in age and gender between the subjects and controls, the Pearson's chi‐squared test and the independent sample *t*‐test were employed. The distribution of genotype and allele frequencies was analyzed using the Pearson's chi‐squared test. Adjustments for age and gender were made through binary logistic regression analysis. Post‐Bonferroni adjustment, a *p*‐value below 0.01 was deemed statistically significant. The comparative risk linked to a primary genotype or allele was calculated using a 95% confidence interval (OR). The genotype's *p*‐value, as shown in the results tables (Table 3 showed the results of the tagSNPs in *B3GNT2* and *GPR35*. Table 4 showed the results of the tagSNPs in *PSMG1*), served to assess the importance of the distribution of genotypes between the case and control groups. Each genotype underwent comparative analysis, with the *p*‐value for each genotype displayed solely when it held significance at the 0.05 threshold. The comparison was made between the severe AS group and the control group, followed by a comparison of the normal AS group with the entire control group. SNPs exhibiting notable variances between AS patients and control groups were linked to their susceptibility to AS. SNPs exhibiting notable variances among patients with severe AS compared to control groups, and between normal AS patients and control groups, were deemed linked to AS severity. Analyzing statistics with the SPSS v.19.0 software package (IBM, Armonk, New York, USA).

**TABLE 3:** shows 5 tagSNPs in *B3GNT2* and the only 1 rs4676410 tagSNP in *GPR35* were compared between all AS patients, severe AS patients, and normal AS patients versus the control subjects. If the *p* < 0.05, the *p*‐value will be highlighted in bold.

SNP		All AS subjects cases/controls		*p*	Severe AS subjects cases/controls		*p*	Normal AS subjects cases/controls		*p*
		Frequencies	OR (95% CI)^c^		Frequencies	OR (95% CI)		Frequencies	OR (95% CI)	
**rs10865331**	All^a^			0.053			**1.053E‐5**			0.164
Genotype	GG	163/164	0.732 (0.511–1.049)		34/164	0.311 (0.188–0.515)	**9.097E‐37**	129/164	1.138 (0.754–1.717)	
	GA	219/245	0.658 (0.469–0.925)	**0.016**	76/245	0.465 (0.303–0.715)	**4.236E‐4**	143/245	0.844 (0.567–1.257)	
	AA	110/81	1^b^		54/81	1		56/81	1	
Allele	G	545/407	1.748 (1.462–2.090)	**8.086E‐10**	144/407	1.102 (0.856–1.418)	0.451	401/407	2.214 (1.809–2.709)	**7.833E‐15**
	A	439/573	1		184/573	1		255/573	1	
**rs4672482**	All			0.689			0.926			0.639
Genotype	AA	165/177	0.860 (0.607–1.220)		57/177	0.909 (0.556–1.487)		108/177	0.837 (0.567–1.236)	
	AG	219/217	0.932 (0.666–1.302)		71/217	0.924 (0.575–1.484)		148/217	0.935 (0.645–1.357)	
	GG	104/96	1		34/96	1		70/96	1	
Allele	A	549/571	1.045 (0.839–1.301)		185/571	0.953 (0.739–1.229)	0.712	364/571	0.905 (0.741–1.106)	0.330
	G	427/409	1		139/409	1		288/409	1	
**rs6545925**	All			**0.017**			**0.023**			0.094
Genotype	CC	131/93	1.454 (1.012–2.090)	**0.043**	48/93	1.652 (1.004–2.716)	**0.047**	83/93	1.360 (0.908–2.037)	
	CG	236/265	0.919 (0.679–1.245)		76/265	0.918 (0.593–1.421)		160/265	0.920 (0.656–1.290)	
	GG	124/128	1		40/128	1		84/128	1	
Allele	C	498/451	1.189 (0.995–1.420)	0.056	172/451	1.274 (0.991–1.637)	0.058	326/451	1.148 (0.941–1.400)	0.172
	G	484/521	1		156/521	1		328/521	1	
**rs4672501**	All			**0.021**			0.482			**0.014**
Genotype	TT	203/182	1.673 (1.151–2.433)	**0.007** *	65/182	1.371 (0.813–2.314)	**9.368E‐10** *	138/182	1.866 (1.211–2.878)	**0.004** *
	TC	221/212	1.564 (1.082–2.260)	**0.017**	72/212	1.304 (0.779–2.183)	**1.378E‐6**	149/212	1.730 (1.129–2.652)	**0.011**
	CC	64/96	1		25/96	1		39/96	1	
Allele	T	627/576	1.260 (1.050–1.512)	**0.013**	202/576	1.161 (0.897–1.503)	0.256	425/576	1.131 (1.069–1.612)	**0.009**
	C	349/404	1		122/404	1		227/404	1	
**rs7605321**	All			0.092			0.214			0.117
Genotype	AA	231/225	0.734 (0.492–1.096)		83/225	0.872 (0.499–1.524)		148/225	0.671 (0.433–1.040)	
	AG	191/213	0.639 (0.426–0.958)	**0.030** ^d^	59/213	0.655 (0.368–1.165)		132/213	0.632 (0.406–0.984)	**0.041** ^d^
	GG	73/52	1		22/52	1		51/52	1	
Allele	A	653/663	0.926 (0.768–1.118)	0.425	225/663	1.044 (0.798–1.367)	0.751	428/663	0.875 (0.710–1.077)	0.207
	G	337/317	1		103/317	1		234/317	1	
**rs4676410**	All			0.385			0.385			**5.217E‐59**
Genotype	GG	235/229	0.821 (0.531–1.270)		77/229	0.740 (0.411–1.332)		8/229	0.009 (0.004–0.020)	**9.926E‐61**
	GA	201/217	0.741 (0.477–1.151)		65/217	0.659 (0.363–1.197)		153/217	0.185 (0.125–0.273)	**9.104E‐19**
	AA	55/44	1		20/44	1		168/44	1	
Allele	A	671/675	0.975 (0.806–1.180)	0.794	219/675	0.942 (0.720–1.233)	0.666	452/675	0.991 (0.801–1.227)	0.937
	G	311/305	1		105/305	1		206/305	1	

^a^ “All” means the *p‐*value that we compare all the three genotypes using the 3 × 2 chi squared method. *p*‐value for individual genotypes is shown only if significant at the 0.05 level.

^b^ The last lines of genotypes or alleles are the minor genotypes or the minor alleles. The other genotypes or alleles are compared to them. The relative risk associated with minor genotypes and minor alleles is estimated as an odds ratio (OR) with a 95% confidence interval (CI).

^c^ OR (95% CI) are adjusted by age and sex using multiple regression analysis.

^d^ *p* < 0.05 but cannot pass Bonferroni correction, which shows marginal significant difference.

* *p* < 0.01 which shows significant difference after Bonferroni correction. When the genotypes or alleles are both related to severe AS and normal AS, they should be considered to be related to severity of AS. If the genotypes or alleles are only related to one of severe AS or normal AS, they should not be considered to be related to severity of AS; we will not use “*” even if *p* < 0.01.

**TABLE 4:** SNPs in *PSMG1* were compared between all AS patients, severe AS patients, and normal AS patients versus the control subjects.

SNP		All AS subjects cases/controls		*p*	Severe AS subjects cases/controls		*p*	Normal AS subjects cases/controls		*p*
		Frequencies	OR (95% CI)^c^		Frequencies	OR (95% CI)		Frequencies	OR (95% CI)	
**rs2142117**	All^a^			0.239			0.169			0.523
Genotype	TT	198/170	1.109 (0.767–1.603)		71/170	1.285 (0.762–2.166)		127/170	1.030 (0.685–1.551)	
	CT	213/232	0.874 (0.611–1.251)		67/232	0.889 (0.529–1.494)		146/232	0.868 (0.584–1.290)	
	CC	84/80	1^b^		26/80	1		58/80	1	
Allele	T	609/572	1.095 (0.914–1.313)	0.325	209/572	1.204 (0.929–1.560)	0.161	400/572	1.046 (0.855–1.280)	0.661
	C	381/392	1		119/392	1		262/392	1	
**rs4816648**	All			**0.001** *			**0.023** ^d^			0.066
Genotype	AA	113/81	2.056 (1.246–3.393)	**0.005** *	36/81	3.111 (1.345–7.195)	**0.006** *	77/81	1.774 (1.032–3.052)	**0.037** ^d^
	AG	342/333	1.514 (0.976–2.347)		120/333	2.523 (1.168–5.446)	**0.015** ^d^	222/333	1.244 (0.774–2.001)	
	GG	38/56	1		8/56	1		30/56	1	
Allele	A	568/495	1.222 (1.021–1.462)	**0.029** ^d^	192/495	1.269 (0.984–1.637)	0.066	376/495	1.199 (0.981–1.465)	0.077
	G	418/445	1		136/445	1		282/445	1	
**rs2837485**	All			0.627			0.638			0.654
Genotype	CC	256/236	0.982 (0.640–1.508)		88/236	1.193 (0.636–2.239)		168/236	0.899 (0.562–1.438)	
	CT	186/194	0.868 (0.560–1.347)		61/194	1.006 (0.527–1.922)		125/194	0.814 (0.503–1.317)	
	TT	53/48	1		15/48	1		38/48	1	
Allele	C	698/666	1.041 (0.857–1.264)	0.686	237/666	1.134 (0.859–1.498)	0.375	461/666	0.999 (0.805–1.239)	0.990
	T	292/290	1		91/290	1		201/290	1	
**rs2837510**	All			0.058			0.652			**0.027** ^d^
Genotype	CC	167/180	1.087 (0.768–1.540)		61/180	0.966 (0.505–1.848)		106/180	1.052 (0.709–1.562)	
	CT	235/193	1.427 (1.020–1.997)	**0.038** ^d^	71/193	0.759 (0.523–1.101)		164/193	1.518 (1.042–2.212)	**0.029** ^d^
	TT	93/109	1		32/109	1		61/109	1	
Allele	C	569/553	1.004 (0.840–1.202)	0.961	193/553	1.063 (0.824–1.370)	0.640	376/553	0.977 (0.800–1.193)	0.820
	T	421/411	1		135/411	1		286/411	1	
**rs2242944**	All			0.119			0.050			0.298
Genotype	GG	176/164	1.393 (0.976–1.987)		69/164	1.911 (1.132–3.226)	**0.014**	107/164	1.185 (0.796–1.765)	
	GA	217/201	1.401 (0.994–1.975)		68/201	1.536 (0.913–2.586)		149/201	1.347 (0.921–0.969)	
	AA	84/109	1		24/109	1		60/109	1	
Allele	G	569/529	1.171 (0.976–1.404)	0.090	206/529	1.407 (1.083–1.826)	**0.010**	363/529	1.069 (0.872–1.310)	0.521
	A	385/419	1		116/419	1		269/419	1	
**rs2150413**	All			0.131			0.481			0.129
Genotype	CC	152/172	1.028 (0.716–1.475)		54/172	1.163 (0.689–1.963)		98/172	0.966 (0.643–1.450)	
	CT	247/218	1.317 (0.937–1.853)		79/218	1.342 (0.817–2.206)		168/218	1.306 (0.894–1.909)	
	TT	86/100	1		27/100	1		59/100	1	
Allele	C	551/562	0.978 (0.818–1.170)	0.809	187/562	1.046 (0.810–1.351)	0.732	364/562	0.947 (0.775–1.156)	0.591
	T	419/418	1		133/418	1		286/418	1	

^a^ “All” means the *p‐*value that we compare all the three genotypes using chi‐squared method. *p*‐value for individual genotypes is shown only if significant at the 0.05 level.

^b^ The last lines of genotypes or alleles are the major genotypes or the major alleles. The other genotypes or alleles are compared to them. The relative risk associated with major genotypes and major alleles is estimated as an odds ratio (OR) with a 95% confidence interval (CI).

^c^ OR (95% CI) are adjusted by age and sex using multiple regression analysis.

^d^ *p* < 0.05 but cannot pass Bonferroni correction, which shows marginal significant difference.

* *p* < 0.01 which shows significant difference after Bonferroni correction. When the genotypes or alleles are both related to severe AS and normal AS, they should be considered to be related to the severity of AS. If the genotypes or alleles are only related to one of severe AS or normal AS, they should not be considered to be related to the severity of AS; we will not use “*” even if the *p* < 0.01.

*p* < 0.05 is defined significant and highlighted in bold.

### 2.6 Declaration of Ethical Standards

In this research, blood specimens from AS patients and control subjects were the remainder of samples for blood routine tests. In gathering and utilizing DNA samples, adherence to clinical data protocols, local Ethics Committee rules, and the 1975 Helsinki Declaration is maintained. All patients and control subjects provided their written, informed agreement (including the parents of these patients or controls if they are younger than 18). Tianjin hospital's Institutional Review Board and the PLA general hospital sanctioned the research methodology.

## 3 Results

### 3.1 Clinical Characteristics

Table 1 displays the BASFI, BASDAI, and mSASSS indices for individuals with AS. In the case of 497 AS patients, the average BASFI stands at 4.296 ± 1.749 (mean ± standard deviation). The average BASDAI stands at 4.195 ± 1.188. The average mSASSS stands at 16.394 ± 16.405. A comparison between patients with severe AS and those with normal AS reveals no notable disparities in terms of gender, age, and the length of the disease (*p* = 0.149, 0.315, 0.810, respectively; Table 2). The severe AS group exhibits a greater BASFI (6.029 ± 2.046) compared to the normal AS group (3.442 ± 0.544) (*p* < 0.001), indicating reduced functionality in the severe AS group. Additionally, the BASDAI values are elevated in the severe AS group (5.488 ± 1.088) compared to the normal AS group (3.559 ± 0.544) (*p* < 0.001), indicating increased disease activity. This also applies to mSASSS (37.073 ± 13.210) in individuals with severe AS compared to 6.210 ± 1.066 in those with normal AS, (*p* < 0.001), suggesting more significant radiographic alterations in severe AS.

### 3.2 Genotype and Allele

To estimate the sample size, we assumed that *p*1 was 0.75, *p*2 was 0.60, and *p* = 0.675. These data were brought into the sample size equation, and the sample size estimate was *n* = 201. Our sample size was more than this. 5 tagSNPs in *B3GNT2* and the only 1 rs4676410 tagSNP in *GPR35* are compared between all AS patients, severe AS patients, and normal AS patients versus the control subjects. Linkage disequilibrium (LD) map of *B3GNT2*, *GPR35*, *PSMG1* comparing different groups were in Figures S2–, S4. When analysing the rs10865331 tagSNP, the GA genotype is reduced (*p* = 0.016), and the G allele is elevated (*p* = 8.086 × 10^−10^). When comparing the severe AS group to the control group, all of them are statistically significant (*p* = 1.053 × 10^−5^) when comparing the severe AS group to the control group. The GG genotype is reduced (*p* = 9.097 × 10^−37^) and the GA genotype is reduced (*p* = 4.236 × 10^−4^). The G allele is elevated (*p* = 7.833 × 10^−15^) when comparing the normal AS group and the control subjects. The rs10865331 tagSNP is related to susceptibility and severity of AS. For rs4672482 SNP, there are no statistical differences in either genotype or allele when comparing between AS (severe & normal) and control groups. When analysing the rs6545925 tagSNP, there is a statistically significant difference in all genotypes when comparing the all AS group and the control group (*p* = 0.017), with an elevated CC genotype (*p* = 0.043). In the comparison of the severe AS group and the control group, there is a statistically significant difference in all genotypes (*p* = 0.023), with an elevated CC genotype (*p* = 0.047). There is no significant difference when comparing the normal AS subjects to controls. The rs6545925 SNP is related to susceptibility to AS; however, this SNP is not related to severity of AS. When analysing the rs4672501 tagSNP, we find more statistically significant results. When comparing the all AS and control groups, the all genotypes are statistically significant (*p* = 0.021), with elevated TT genotypes (*p* = 0.007), elevated TC genotypes (*p* = 0.017), and elevated T alleles (*p* = 0.013); and when comparing the severe AS and control groups, the TT genotypes are elevated (*p* = 9.368 × 10^−10^), with elevated TC genotypes (*p* = 1.378 × 10^−6^). When comparing the normal AS group to the control group, all genotypes are statistically significant (*p* = 0.014), with elevated TT genotype (*p* = 0.004), elevated TC genotype (*p* = 0011), and elevated T allele (*p* = 0.009), suggesting that the rs4672501 SNP is associated with susceptibility and severity of AS. When analysing the rs7605321 tagSNP, we find a decrease in AG genotype when comparing the all AS group to the control group (*p* = 0.030), and a decrease in AG genotype when comparing the all AS group to the control group (*p* = 0.041); however, both of which can only be considered marginally significant after Bonferroni correction. When analysing the rs4676410 SNP, we only find significant difference when comparing the normal AS and control groups (*p* = 5.217 × 10^−59^), with a significant decrease in the GG genotype (*p* = 9.926 × 10^−61^), and a significant decrease in the GA genotype (*p* = 9.104 × 10^−19^). Overall, among the five selected tagSNPs of *B3GNT2*, the rs10865331, rs6545925, and rs4672501 tagSNP are associated with the susceptibility to AS. Additionally, for the first time, we find that the rs4672501 SNP is not only associated with the susceptibility to AS, but also associated with the severity of AS. For the *GPR35* gene, most of the SNPs are in high linkage disequilibrium; therefore, only one tagSNP: the rs4676410 SNP is selected after haploview4.2 software calculation. The results of the analysed experiments suggested that there was 1 positive result when comparing the normal AS group and the control group, and there is a statistically significant result when comparing all genotypes (*p* = 5.217 × 10^−59^), and the GG genotype significantly lower (*p* = 9.926 × 10^−61^); and GA genotype significantly lower (*p* = 9.104 × 10^−19^). Thus the rs4676410 SNP on *GPR35* gene is associated with susceptibility to AS, but not with severity of AS.

After analyzing the six tagSNP loci in *PSMG1*, we find that the rs2142117 SNP is not associated with either AS susceptibility or severity. In contrast, the rs4816648 SNP shows a clear correlation. When comparing all AS patients to controls, all genotypes were statistically significant (*p* = 0.001), with a significant rise in the AA genotype (*p* = 0.005) and a significant rise in the A allele (*p* = 0.029). When comparing the severe AS group to the control group, all genotypes are statistically significant (*p* = 0.023), with a significant rise in the AA genotype (*p* = 0.006) and AG genotype (*p* = 0.015). When comparing the normal AS group to the control group, the AA genotype rises (*p* = 0.037). Therefore, we find for the first time that the rs4816648 SNP is associated with both susceptibility and severity of AS. We analyze the rs2837485 SNP; however, fInd no statistical significance when comparing between groups. Therefore, the rs2837485 SNP is not associated with AS susceptibility and severity. We analyze the rs2837510 SNP and fInd that CT genotypes are elevated when comparing all AS patients with controls (*p* = 0.038) and when comparing patients in the normal AS group with controls (*p* = 0.029); however, the *p*‐values for these findings are between 0.01 and 0.05, and after Bonferroni correction, these loci are only considered to be borderline relevant. For the rs2242944 SNP, a rise in GG genotype (*p* = 0.014) and an elevated G allele (*p* = 0.010) are found when comparing the severe AS group to the control group. After Bonferroni correction, these loci are only considered borderline relevant. For the rs2150413 SNP, there is no statistical difference in the comparisons between the data groups, and therefore the rs2150413 SNP is not associated with AS susceptibility or severity.

## 4 Discussion

The enzyme B3GNT2, a member of the β‐1,3‐N‐acetylglucosaminyltransferases (B3GNT) family, aids in the transport of N‐acetylglucosamine (GlcNAc) via a β‐1,3‐bond (Narimatsu 2006). Enzyme B3GNT2 plays a key role in initiating and elongating polylactosamine chains, particularly focusing on the terminal disaccharide unit, thereby facilitating the lengthening of polylactosamine chains of different lengths (Kadirvelraj et al. 2021; Hao et al. 2021). Studies encompassing the entire genome (GWAS) indicated a link between the *B3GNT2* gene and the susceptibility to AS in Caucasian groups (Australo‐Anglo‐American Spondyloarthritis et al. 2010). In patients with psoriasis and rheumatoid, there is a decrease in *B3GNT2* gene activity, linking *B3GNT2* to various autoimmune disorders (Tsoi et al. 2012; Okada et al. 2012). Mice deficient in *B3GNT2* show a significant reduction in polylactosamine in N‐glycans and increased activity in T cells, B cells, and macrophages (Togayachi et al. 2007). According to our findings, the *B3GNT2* gene is linked to both the susceptibility to AS and the severity of AS. Echoing research on psoriasis and rheumatoid conditions, reducing B3GNT2 levels in AS patients impacts the survival of T cells, B cells, and macrophages. Ultimately, this influences the beginning and intensity of AS. The outcomes of our study corroborate earlier GWAS outcomes in populations from the west.

GWAS additionally identified disease‐associated polymorphisms in the coding and inter‐gene regions surrounding *GPR35*. Research indicates that *GPR35* could be a major factor in conditions like inflammatory pain, asthma, diabetes, hypertension, heart disease, and IBD (Mackenzie et al. 2011). A genetic alteration in *GPR35* (rs3749171, resulting in a T108M change) is associated with primary sclerosing cholangitis (PSC) and ulcerative colitis (UC) risk (Ellinghaus et al. 2013; Ji et al. 2017). *GPR35* controls the growth of intestinal epithelial cells by activating Src phosphorylation via its Na/K‐ATPase interaction (Schneditz et al. 2019). The discharge of enhancers and altered activation of *GPR35* leads to the growth and movement of gastric cancer cells, a notable reduction in certain immune cells (CD8 + T cells and CD4 + memory T cells), and/or infiltration (T‐cells and macrophages). Concurrently, elevated *GRP35* levels result in unfavorable outcomes for gastric cancer sufferers, partly due to their role in enhancing the immune system's penetration of macrophages, subsequently triggering the polarization of M2 macrophages (Shu et al. 2022). The activation of *GPR35* is both essential and adequate for safeguarding against KynA ischemia. Upon attachment to KynA, *GPR35* triggers Gi and G12/13 coupling signals, leading to its transport to the outer mitochondrial membrane, where it forms a substantial and indirect bond with ATP Synthase inhibitory Factor Subunit 1 (ATPIF1). The activation of *GPR35* triggered the dimerization of ATP synthase in a manner reliant on ATPIF1 and sensitive to pertussis toxin, thereby inhibiting the loss of ATP due to ischemia (Wyant et al. 2022). Human immune cells, such as monocytes (CD14+), T cells (CD3+), neutrophils, and a range of dendritic and natural killer T cells (CD56+) (Quon et al. 2020), exhibit *GPR35* expression. Recent studies using single‐cell RNA sequencing on immune cells in the lamina propria and Peyer's patch cells in the mouse small intestine revealed a predominant presence of GPR35 in clusters of dendritic cells (CD103+ CD11b‐) and macrophages, along with tiny, 100‐cell “unresolved” clusters (Xu et al. 2019). According to our findings, the rs4676410 SNP in the *GPR35* gene correlates with an increased risk of AS, though it does not correlate with the intensity of AS. Given these findings, it's plausible to infer that *GPR35* impacts monocytes, T‐lymphocytes, neutrophils, and NK‐cells, thereby influencing the development of AS. Consequently, our research indicates that *GPR35*'s influence on the inflammatory reaction in IBD is also relevant to AS.

Recent studies indicate that *PSMG1* heightens the risk of developing inflammatory bowel diseases (Waterman et al. 2011; Latiano et al. 2011; Wagner et al. 2010). It was discovered that *PSMG1* plays a role in cellular growth (Vidal‐Taboada et al. 2000; Song et al. 2008). Additionally, the researchers found *PSMG1* plays an important role in cancer development and underscored the significance of the miR‐484–*PSMG1* pathway in prostate cancer (Lee et al. 2020). Fang et al. discovered an increase in *PSMG1* levels in COVID‐19 patients who had been discharged and tested positive once more (Fang et al. 2022). The products of *PSMG1* are involved in the maturation of proteasomes, differentiation of macrophages, control of megakaryocytic gene activity, development of T cells, and the phenotypic transition of hematopoietic cells from erythrocyte to megakaryocytic growth (Tajuddin et al. 2016). The impact of *PSMG1* on amino acid alterations is due to positive selection. Consequently, it serves as a valuable tool for subsequent research, mechanically connecting genes and amino acids to proactively determine aging and lifespan (Sahm et al. 2018). According to our studies, the rs4816648 SNP in the *PSMG1* gene correlates with the vulnerability and intensity of ankylosing spondylitis. In the case of an inflammatory disorder like AS, the ability of *PSMG1* to affect both the susceptibility and severity of AS is expected, considering the gene's capacity to impact the functioning and differentiation of macrophages and T cells. This study has potential limitations: tagSNPs selected using the principle of linkage disequilibrium can only substitute for gene correlations to a certain extent, and the test efficacy cannot reach the level of GWAS. If available, it would be better to choose the GWAS method to conduct replicated studies in different populations, which will be more convincing. In addition, the method of grouping AS patients using surgical indications has limitations, and the criteria for grouping as well as the accuracy of the grouping depend on the clinical experience of rheumatologists. Our study finds an association between these three genes and AS in the Chinese Han population, corroborating findings in western populations. This suggests that these three genes are universal in both western and eastern populations. This provides an important basis for the study of the etiology of AS.

## Author Contributions

**Zijian Lian:** conceptualization, data curation, methodology, writing – original draft. **Bin Zhao:** conceptualization, investigation, methodology, writing – review and editing. **Wei Luo:** data curation. **Jun Liu:** data curation. **Jing Wang:** data curation. **Wei Chai:** funding acquisition. **Yan Wang:** funding acquisition. **Songqing Ye:** writing – review and editing. **Xinlong Ma:** funding acquisition, validation, writing – review and editing.

## Conflicts of Interest

The authors declare no conflicts of interest.

## Supporting information


**Figure S1:** The positions of each selected tagSNP on the genes. The SNP rs4672482 is in the exon 2 of *B3GNT2*. The SNP rs4676410 is in the exon 6 of *GPR35*. The SNP rs2242944 is near the promoter of *PSMG1* gene. Other SNPs are all in the introns of their respective genes.

**Figure S2:** Linkage disequilibrium (LD) map of *B3GNT2*, *GPR35*, *PSMG1* comparing All AS patients and controls. Darker color indicates higher linkage disequilibrium (LD), lighter color indicates less LD. Numbers in the squares indicate correlation coefficient (R2) value.

**Figure S3:** LD map of *B3GNT2*, *GPR35*, *PSMG1* comparing severe AS patients to controls.

**Figure S4:** LD map of *B3GNT2*, *GPR35*, *PSMG1* comparing normal AS patients to controls.

## Data Availability

The data that support the findings of this study are available on request from the corresponding author. The data are not publicly available due to privacy or ethical restrictions.
