# Supplementary figures and images for: B3GNT2 , GPR35 , PSMG1 Gene Polymorphisms Are Related With Susceptibility and Severity of Ankylosing Spondylitis in Chinese Han Population
Source: Mol Genet Genomic Med. 2025 Jul 30;13(8):e70125. doi: 10.1002/mgg3.70125 (PMC12308515; doi:10.1002/mgg3.70125)

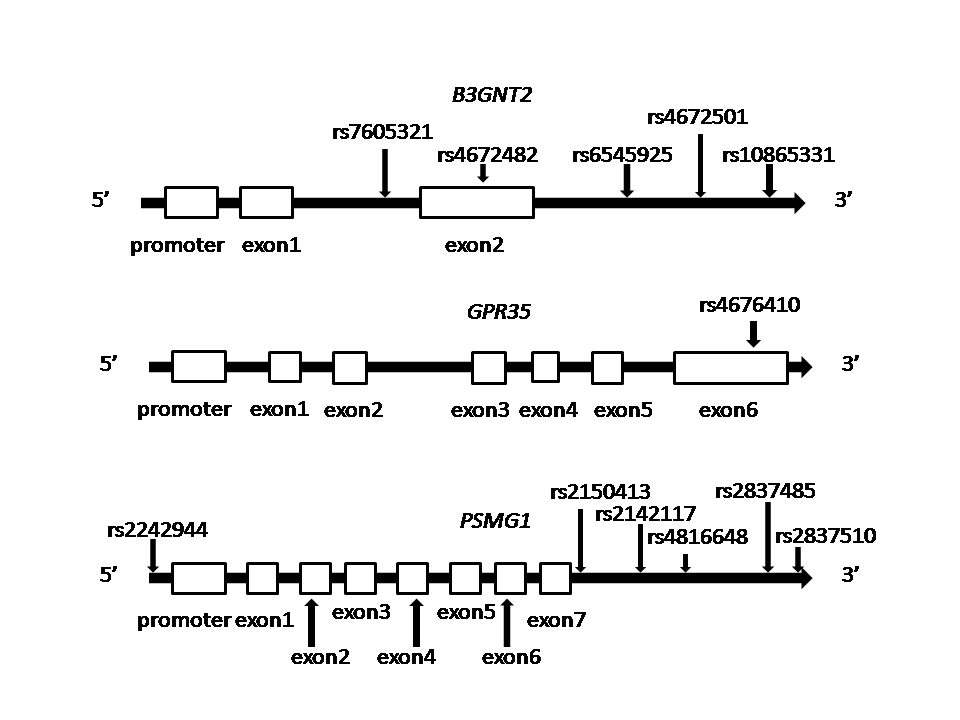

Supplement: Supplementary file 1 — Figure S1: The positions of each selected tagSNP on the genes. The SNP rs4672482 is in the exon 2 of B3GNT2. The SNP rs4676410 is in the exon 6 of GPR35. The SNP rs2242944 is near the promoter of PSMG1 gene. Other SNPs are all in the introns of their respective genes. [file MGG3-13-e70125-s004.tif]

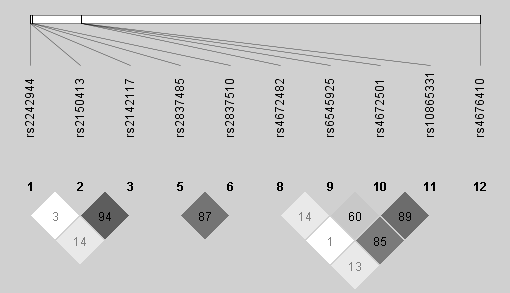

Supplement: Supplementary file 2 — Figure S2: Linkage disequilibrium (LD) map of B3GNT2, GPR35, PSMG1 comparing All AS patients and controls. Darker color indicates higher linkage disequilibrium (LD), lighter color indicates less LD. Numbers in the squares indicate correlation coefficient (R2) value. [file MGG3-13-e70125-s003.png]

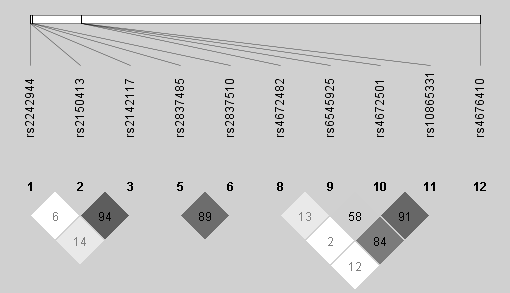

Supplement: Supplementary file 3 — Figure S3: LD map of B3GNT2, GPR35, PSMG1 comparing severe AS patients to controls. [file MGG3-13-e70125-s001.png]

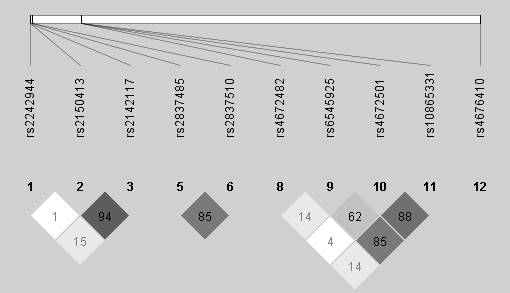

Supplement: Supplementary file 4 — Figure S4: LD map of B3GNT2, GPR35, PSMG1 comparing normal AS patients to controls. [file MGG3-13-e70125-s002.png]
